# Identity work of public hospital nurses during the COVID-19 pandemic in South Africa

**DOI:** 10.4102/curationis.v47i1.2506

**Published:** 2024-08-08

**Authors:** Nosipho M. Maseko, Roslyn T. De Braine

**Affiliations:** 1 Department of Industrial Psychology and People Management, College of Business and Economics, University of Johannesburg, Johannesburg, South Africa

**Keywords:** COVID-19, identity work, meaningful work, nurses, hospitals, experiences, calling

## Abstract

**Background:**

Nurses play a remarkable role in our healthcare system and contribute to the wellbeing of communities at large. During the coronavirus disease 2019 (COVID-19) pandemic, nurses faced various challenges to provide adequate patient healthcare.

**Objectives:**

This study aimed to explore the identity work of public hospital nurses during the COVID-19 pandemic.

**Method:**

The study followed a phenomenological qualitative approach with an interpretive view, employing two sampling methods: purposive and snowball sampling. The sample comprised 11 nurses from a public hospital in the Gauteng province. The data were collected using semi-structured interviews and analysed using thematic analysis.

**Results:**

The findings revealed that the nurses faced identity demands, which resulted in them experiencing identity tensions. There was also a need for recognition and support; their work served a greater purpose and was meaningful to them. The nurses used different identity work strategies, such as family support, spiritual upliftment and meaningful work to deal with the identity tensions and demands they experienced.

**Conclusion:**

Strategies such as counselling and wellbeing programmes should be implemented to assist nurses in dealing with the physical and psychological effects of working in the health sector during pandemics and epidemics. Hospitals and governments should create healthier working environments by conducting workshops, training and upskilling initiatives, encouraging nurses’ inclusion in policymaking and implementation.

**Contribution:**

The study provided insight into the challenges nurses encountered during the COVID-19 pandemic, how these challenges affected their nursing identity and roles, and the strategies they used to maintain their sense of self in their work.

## Introduction

In various studies on identities in organisations, identity work has emerged as an important concept. Individuals often engage in identity work during transitions and change, particularly when they feel a threat to their identities (Caza, Vough & Puranik 2018:900); identity work involves:

[*C*]ognitive, discursive, physical, and behavioural activities that individuals undertake with the goal of forming, repairing, maintaining, strengthening, revising, or rejecting collective, role, and personal self-meanings within the boundaries of their social contexts. (p. 895)

One context that brought significant change and transition and a tremendous amount of strain on already overwhelmed healthcare systems and health workers worldwide was the coronavirus disease 2019 (COVID-19) pandemic (Kaye et al. 2021:297; Muller et al. 2020:113440). In particular, the pandemic hugely impacted nurses and their work environments. Situational factors such as additional workload, long hours and shortages in personal protection equipment (PPE) led to an increase in the physical and mental demands placed on nurses, which led to higher stress levels and post-traumatic stress symptoms (Jansoon & Rello 2020:1). Nurses’ psychological states were significantly affected (Arasli et al. 2020:2). Consequently, the nurses experienced changes in how they fulfilled their nursing roles and, additionally, had to learn how to work wearing masks, which was previously only required in certain situations. In addition, nurses experienced uncertainty, social stigma, physical and psychological distress (Almomani et al. 2022:1). Caring for sick patients is often associated with emotional exhaustion and stress (Manyisa & Van Aswegen 2017:29). The wellbeing of nurses is influenced by many factors, such as having to deal with role ambiguity, emotional demands and increased workload (Patience, De Braine & Dhanpat 2020:413). Situational factors external to the individual that affect an individual’s response to creating balance in their lives are regarded as identity demands (Kreiner, Hollensbe & Sheep 2006:1034). Examples of situational factors could be increased workload and long hours. Identity tensions are experienced by individuals because of identity demands (Kreiner et al. 2006:1034). Identity work involves balancing and resolving the tensions between the self and the demands of the work environment in which the individual’s identity is negotiated (Adams & Crafford 2012:2). Caza et al. (2018:904) further indicated the need to explore identity work across various organisational contexts, particularly to close the gap in knowledge on identity work in Africa, Asia and South America. Not much is known about the effects of the pandemic on nurses’ identity work in South Africa. This article focuses on how the COVID-19 pandemic, as a specific event, led to transitions and strain, creating identity demands that led to identity tensions and identity work in nurses during the pandemic.

## Research methods and design

### Design

A phenomenological qualitative approach was adopted to understand better nurses’ experiences and perceptions during the COVID-19 pandemic and to determine their strategies for remaining committed to their jobs and maintaining their identities. The main foundation of phenomenology is to understand the lived experiences of others (Van Manen 2017:811). An interpretivist philosophy was used, focusing on subjective and descriptive methods of scientific inquiry (Al-Ababneh 2020:6) to understand and explain human and social reality.

### Study setting and participants

The research was conducted at a public hospital in Gauteng province, South Africa. The first author negotiated access to the nurses with the assistance of the hospital social worker. Purposive sampling was used to get the first participants, based on their relevance to the research questions and shared experiences of the pandemic. Thereafter, snowball sampling was used to get additional participants after the initial participants started to refer or invite other participants. Snowball sampling is based on a chain reaction process, where a chosen participant will either invite or refer someone else to participate in the study. Eleven nurses from six wards in the hospital who were able to use Zoom or were available to meet face-to-face were interviewed by the first author, using semi-structured interviews. Because of the open-ended nature of questions in semi-structured interviews, the authors can revise and allow the emergence of new questions in order to derive a more in-depth understanding during an interview of participants’ lived experiences. Data saturation was eventually reached based on the data received from all the participants and the similarity in the responses.

### Data collection

The data for this study was collected by the first author through semi-structured interviews comprising open-ended questions. This study’s aim was to examine the identity work of nurses in public hospitals in South Africa by finding out what challenges were faced when trying to perform their duties during the COVID-19 pandemic, what influenced their identity construction and which identity work strategies nurses in public hospitals used (please refer to Appendix 1: Semi-structured Interview Guide).

The first author set up interview appointments with each participant, and interviews were conducted during the nurses’ lunch breaks or on their off days to not interfere with their work schedules. Each interview lasted between 30 and 45 min. Owing to the COVID-19 pandemic crisis and social-distancing measures in place at the time, some interviews were conducted via Zoom. Once a time and date were agreed upon with each participant, the first author sent an invitation with the online meeting link to the participant. No interview commenced before the participant had signed and returned the consent form. Those who were interviewed via Zoom sent their signed forms back via email.

All interviews were recorded for later transcription. The measures for handling the recordings and keeping them safe were discussed with the participants. The participants granted their permission for the interviews to be recorded (nine for the face-to-face and two for online interviews). To maintain the participants’ anonymity, participant numbers were assigned to each interviewee rather than using their names.

### Data analysis

Thematic analysis was used to analyse the interview data. This method allows researchers to group the information into themes and sub-themes (Braun & Clarke 2006:81). To summarise and make sense of the interview transcripts and notes, the six phases of thematic analysis by Braun and Clarke (2006:87) were followed: (1) through reading and re-reading, familiarisation of the interview transcripts was made; (2) initial codes were generated based on commonality; (3) searching for initial themes involved grouping the codes into potential themes, and codes were then reviewed and combined to develop themes and sub-themes; (4) the themes were then reviewed in relation to the information that was extracted from the coding of the entire data-set in which a thematic map of analysis was generated; (5) each theme and sub-theme was analysed and some were also renamed; (6) lastly, the data were reviewed, the themes revisited, a final thematic map drafted, and the importance and meaning of each theme, and its links to the themes were documented. Extracts were selected to substantiate the themes created. Themes were identified, and these themes were linked to identity work tactics and strategies found in existing literature.

### Ethical considerations

The Research Ethics Committee of the Department of Industrial Psychology and People Management (IPPM), University of Johannesburg, granted ethical clearance for this study (ethical clearance code IPPM-2021-472[M]). Informed consent was obtained in writing from all participants in the study. The first author explained the purpose of the study and the data collection process to the participants. As participation was voluntary, participants were informed of their right to withdraw from the study at any time. No personal questions or identifying particulars were asked. The confidentiality of the participants was maintained by not revealing their names during the data collection, analysis or reporting of the findings.

## Results

The demographic makeup, including gender, tenure, age, occupation, and department of the sample, is displayed in Table 1.

**TABLE 1:** Demographic data of research participants.

Participant ID	Age (years)	Gender	Occupation	Tenure (years)	Department
1	37	Female	Professional nurse	13	Medical ward
2	45	Female	Professional nurse	18	Medical ward
3	53	Female	Professional nurse	21	Respiratory ward
4	36	Female	Assistant and Auxillary nurse	5	Medical ward
5	39	Female	Professional nurse	7	ICU ward
6	40	Female	Professional nurse and midwife	12	Obstetrics and gynaecology ward
7	56	Female	Professional nurse	22	Paediatrics ward
8	60	Female	Professional nurse	31	Maternity ward
9	62	Female	Clinical nurse practitioner	27	TB or HIV clinic
10	42	Female	Professional nurse	9	TB or HIV clinic
11	64	Female	Professional nurse	47	TB or HIV clinic

HIV, human immunodeficiency virus; ICU, intensive care unit; TB, tuberculosis.

A total of 11 participants were interviewed. The participants were all professional female nurses in their mid-30s to early 60s with a strong tenure in different fields.

The findings from the participants were categorised according to the themes and sub-themes, as shown in Table 2.

**TABLE 2:** Themes and sub-themes.

Themes		Sub-themes
1.	Reasons for choosing nursing as a profession	1.1 Early age desire 1.2 Place of compassion for others 1.3 Desire to help others
2.	Roles and duties of nurses	2.1 Producing reports and working on statistics; taking doctors’ orders 2.2 Providing patient care.
3.	Feelings brought on by COVID-19	3.1 Feelings of fear 3.2 Feelings of uncertainty 3.3 Feelings of betrayal 3.4 Trauma of losing patients
4.	Nurses’ personal challenges and experiences as a result of COVID-19	4.1 Feelings of exhaustion and burnout 4.2 High stress levels 4.3 Community ties 4.4 Emotional and mental demands 4.5 Unsafe working conditions 4.6 Work–life balance 4.7 Patients not being able to see loved ones
5.	Challenges faced by the hospitals as a result of COVID-19	5.1 Equipment and staff shortages 5.2 Lack of social distancing 5.3 Lack of recognition
6.	Changes in how nurses fulfilled their roles and duties in COVID-19	6.1 Changes in the work environment 6.2 Having to upskill and learn new tasks
7.	Nurses’ sources of social support	7.1 Spiritual upliftment 7.2 Family support
8.	Strategies and tactics followed by the nurses	8.1 Adapting 8.2 Resisting 8.3 Avoiding 8.4 Recognition (dawning)
9.	Purpose	9.1 Calling 9.2 Meaningful work

### Theme 1: Reasons for choosing nursing as a profession

Some of the nurses chose nursing as a career from an early age desire to become a nurse. They also chose nursing from a place of compassion for others and a desire to help others. Others mentioned that nursing was always their first choice even after pursuing other career options. The following extract highlights why one participant chose nursing:

‘So, I grew up in the rural areas and as you know, back then nurses were scarce in the rural areas. So even before I actually went into nursing, my mom used to tell me how I would like to play nurse when playing with other kids and treat them and even the outfits I did for my dolls was nurse outfits. Nursing for me is not just a job; I love it and am very passionate about it.’ (Participant 3, 53 years old, professional nurse)

### Theme 2: Roles and duties of nurses

The second theme that emerged was the roles and duties of the nurses. This question was asked in the interviews to understand the nurses’ work, which would help provide a better understanding of their identity work and their typical day. These tasks included producing reports, working on statistics, taking the doctors’ orders and providing patient care. The following extract illustrates one participant’s typical day and the many varied tasks she performed:

‘We come in and take a report around 7:00, 7h30 then we go to the assembly, and we give each person a report. Thereafter we feed the patients then give them medication, then the doctors arrive, and they do their rounds at 9:00, 9:30, depends on how quick the doctor is. Around three in the afternoon is when we prepare the patients for visiting hours and seeing their relatives, then after 4 we wrap.’ (Participant 1, 37 years old, professional nurse)

### Theme 3: Feelings brought on by COVID-19

From the data, the theme that emerged was the feelings that COVID-19 brought on for the nurses. The following sub-themes were feelings of fear, uncertainty, betrayal and trauma from losing patients.

#### Sub-theme 3.1: Feelings of fear

Some of the nurses spoke of how the pandemic brought about feelings of fear for them. These included not knowing whether they were infected and the fear of being infected. This is illustrated in the extract below:

‘It has changed it a lot because now you are nursing the patient with fear especially since the results only came later on in the day. So now you have been nursing someone only to find later on in the day that they are positive, meanwhile you did not have your full PPE on. Now you start thinking, “am I positive or negative?” and you were busy treating other patients.’ (Participant 4, 36 years old, assistant or auxiliary nurse)

#### Sub-theme 3.2: Feelings of uncertainty

The pandemic also brought about much uncertainty for the nurses, especially concerning what they should do and how to react, because not much was known about the virus:

‘It was fearing what we did not know, we didn’t know what to do, and didn’t know much about it first of all.’ (Participant 4, 36 years old, assistant or auxiliary nurse)

#### Sub-theme 3.3: Feelings of betrayal

One of the nurses mentioned that the constantly changing statistics and data about COVID-19 brought about feelings of betrayal, as illustrated in the extract below:

‘In my opinion I feel like this situation betrayed the nurses. Because it feels like you are saying this but the results become something else.’ (Participant 1, 37 years old, professional nurse)

#### Sub-theme 3.4: Trauma of losing patients

The nurses were profoundly affected by the sudden loss of patients from COVID-19. Having to deal with rising death rates daily left some of the nurses traumatised. The extract below illustrates this study’s findings:

‘People were dying as well, you would be on night shift and next thing five people gone, it was traumatising and then you go to the mortuary and you find it full also. You know what was really traumatising, wrapping the patients. At some point, it felt like we were working at fish and chips and just wrapping them one after the other.’ (Participant 4, 36 years old, assistant or auxiliary nurse)

### Theme 4: Nurses’ personal challenges and experiences as a result of COVID-19

Some of the personal challenges nurses experienced included long working hours and burnout, high stress levels, having ties to the community yet not being able to properly care for patients they knew, emotional and mental demands, unsafe working conditions, work–life balance and the burden of patients not being able to see loved ones.

#### Sub-theme 4.1: Feelings of exhaustion and burnout

Some of the nurses mentioned that working long hours led to their being left exhausted and burnt out:

‘The working hours and the shortage of staff, the ratio of us to patients is not enough. They are bringing in contract workers but some of them know nothing so it’s difficult to teach people when there is a lot of work to get through.’ (Participant 8, 60 years old, professional nurse)

#### Sub-theme 4.2: High stress levels

The increase in workload owing to the demands of the job, dealing with more patients and other staff members being infected resulted in an increase in stress levels for the nurses, as illustrated by the following extract:

‘Our labs were suffering from heavy backlog, this resulted in us delaying and having high workload, and not being able to move forward with treatment. The workload is a lot, we are nursing more than we used to and the current hospital renovations we have has made things much more difficult, it made the working environment much more hectic.’ (Participant 1, 37 years old, professional nurse)

#### Sub-theme 4.3: Community ties

Having ties to the community made the situation incredibly challenging for some nurses. Because many grew up or lived in the area where they worked; most of the people they treated were either neighbours or close family friends:

‘I think for me the biggest challenge with working here is that I grew up here and I work here so I know everyone. If I don’t know you personally, I know your dad or sisters and as a patient it’s very hectic.’ (Participant 1, 37 years old, professional nurse)

#### Sub-theme 4.4: Emotional and mental demands

The COVID-19 pandemic brought on many emotional and mental demands for the nurses, and, as a result, some found themselves being more emotional in certain situations and others struggling mentally. The extract below supports our findings:

‘It has affected me a lot and personally, as well, we had to find different ways to cope with our work and emotionally. COVID-19 is not something we were maybe taught at school, so it was unknown, and it has challenged us in every way. We were taught to be strong for the patient and not to be seen crying in front of them, but we found ourselves breaking down in front of them because this was beyond us.’ (Participant 5, 39 years old, professional nurse)

#### Sub-theme 4.5: Unsafe working conditions

Treating infected patients and working while tired meant that the nurses were working in unsafe conditions and exposing themselves to danger, as illustrated by the extract below:

‘Many of the nurses were exposed to the virus because we were treating infected patients, making us high risk. You arrive here exhausted, and you are exposed to a lot of things and infections.’ (Participant 2, 45 years old, professional nurse)

#### Sub-theme 4.6: Work–life balance

Some of the study participants mentioned a need to find a balance between work demands and home life. Some of the nurses mentioned how, in order to get a balance between work and home, they left work issues at work before heading home and vice versa:

‘When you are a nurse, you have to leave your problems at the gate, you become brave and develop a thick skin.’ (Participant 7, 56 years old, professional nurse)

#### Sub-theme 4.7: Patients not being able to see loved ones

Patients not being able to see their loved ones led to their being depressed and lonely, which meant that the nurses had to provide the emotional support that they would usually get from loved ones, which affected the nurses. The following extract illustrates this:

‘It affected me a lot, especially the lockdown, since relatives could not come and see their family members. It was extremely hard for me to see people receive a call that your mom and dad is no longer, and that time they will ask “how did this happen?” because they did not see this person’s condition change. That affected me.’ (Participant 4, 36 years old, assistant or auxiliary nurse)

### Theme 5: Challenges faced by the hospitals as a result of COVID-19

#### Sub-theme 5.1: Equipment and staff shortages

The COVID-19 pandemic brought about many challenges for the hospitals. They experienced shortages of equipment such as masks, PPE and oxygen. When staff became infected and had to quarantine, this would lead to shortages in staff:

‘You would arrive here and find there are not any N95 masks and have to make use of normal surgical masks, and those are not as efficient. And then also the equipment; we do not have enough oxygen, and we would have to use cylinders. When some staff were in quarantine and they cannot come back until that is over, we would have shortage of staff, which means increased workload.’ (Participant 2, 45 years old, professional nurse)

#### Sub-theme 5.2: Lack of social distancing

Some hospital wards were not built to accommodate as many patients as they did during the pandemic. The increase in number of patients resulted in overcrowding in the wards, which meant that proper social distancing protocols could no longer be observed:

‘The wards get full, we have no space for [*the patients*] to sleep and then we have to make them sleep on stretchers because we do not have beds. We end up now not being able to social distance properly because the ward becomes full.’ (Participant 3, 53 years old, professional nurse)

#### Sub-theme 5.3: Lack of recognition

Many of the nurses spoke of recognition and acknowledgement. They stated that having managerial support or being valued in some way would have made a significant difference, especially during this time. The following extract illustrates the need for recognition and incentives:

‘The painful thing is people do not see the work we do for them or acknowledge it. We work for people who do not have a thank-you but because we love what we do and nursing, it does not deter us from helping people.’ (Participant 2, 45 years old, professional nurse)

### Theme 6: Changes in how nurses fulfilled their roles and duties in COVID-19

How nurses fulfilled their roles and duties changed during COVID-19. The pandemic forced nurses to change the way they work and their working environments. In addition, some nurses had to upskill themselves and learn new tasks outside their normal duties to assist in other wards.

#### Sub-theme 6.1: Changes in the work environment

Some participants described the changes in the working environment as a result of COVID-19, especially regarding protective equipment and exercising additional caution:

‘Now we work with masks around the clock, whether it is hot or not.’ (Participant 3, 53 years old, professional nurse)

#### Sub-theme 6.2: Having to upskill and learn new tasks

The pandemic also brought with it the need to upskill and learn new tasks during an already stressful period, as illustrated in the following extract:

‘We also had to refresh our knowledge on other skills we were not using anymore because it isn’t in your skill set but because of the situation you had to learn about vents because now the ICU was packed.’ (Participant 5, 39 years old, professional nurse)

### Theme 7: Nurses’ sources of social support

During the pandemic, some of the nurses found support from different facets of their lives (spiritual upliftment and family support) to better cope with the demands of the pandemic, as illustrated below.

#### Sub-theme 7.1: Spiritual upliftment

Some nurses found strength in their faith and religious beliefs:

‘Emotionally we have adapted to more spiritual upliftment, now we pray before shift depending on our individual beliefs, also open bible verses, and just encourage each other.’ (Participant 5, 39 years old, professional nurse)

#### Sub-theme 7.2: Family support

For some, the importance of family as a support structure was highlighted during the pandemic:

‘I enjoy working in groups and spending time with friends and family especially now, after COVID-19.’ (Participant 9, 62 years old, clinical nurse practitioner)

### Theme 8: Strategies and tactics used by the nurses

In this study, we identified four identity-work tactics used by the nurses to deal with the demands of the COVID-19 pandemic: adapting, resisting, avoiding and recognition (dawning). These are discussed below.

#### Sub-theme 8.1: Adapting

Some of the nurses in this study found themselves having to adapt to the situation to be able to help during this time, even though they were not comfortable with the changes, as illustrated in the extract below:

‘For me personally it was the fact that I was expected to work with any group of people and I prefer to work with adult males, and with COVID-19 I was now in female wards and then with babies and there was no preference; you had to work with what you were given. I was forced to adjust; one day you are in this environment and the next day you come back to work and find all of that has changed.’ (Participant 5, 39 years old, professional nurse)

#### Sub-theme 8.2: Resisting

The nurses in this study also showed some resistance to the change that came with working during a pandemic and dealing with an unknown virus:

‘On one occasion after our first case, I was in charge when our matron was not around, and I came in to work to find them [*nurses*] all outside refusing to go inside because we were scared and did not want to go in until it was fumigated. I was there running around trying to call the matron and CEO, and at that time the patients were already inside waiting to be treated but also could see things are not happening as usual. And you know when the CEO finally arrived that day, he threatened to fire all of us for not working. [*And this was justified because no one wants to work in an environment where you are scared, because when you scared that is when mistakes happen because you are not focused on what you are doing?*] Yes, and the patients can sue you.’ (Participant 11, 64 years old, professional nurse)

#### Sub-theme 8.3: Avoiding

To better deal with some of the challenges that came about because of the pandemic, some nurses chose to avoid certain situations that would put them in uncomfortable positions or make them untrue to themselves:

‘You have those days were some patients try and test you or challenge you. In these situations, you just walk away and ask someone else to please take over from you.’ (Participant 8, 60 years old, professional nurse)

#### Sub-theme 8.4: Recognition (dawning)

This study found that some of the nurses reached a point of recognition (dawning) that the situation had changed, and things were different, which meant that they needed to change how they reacted to and handled matters:

‘It has been hard but what more can we do, there is nothing we can do we chose to do this job so what else can be done.’ (Participant 3, 53 years old, professional nurse)

### Theme 9: Purpose (calling) and meaningful work

In presenting the findings, the first question posed was why the nurses chose the nursing profession to begin with. To get a sense of the nurses’ identity work, the different roles and duties they performed daily were examined. This was followed by describing the different feelings brought on by COVID-19 and the various challenges experienced both by the nurses and the hospitals. These challenges changed their way of working; nurses drew strength from different facets of their lives and used certain tactics to better deal with all the challenges they experienced from the pandemic. The main finding in this study was that nursing was more than simply a job; it was a calling with a sense of purpose for the nurses. They placed more importance on their work and serving their communities even during a pandemic and believed this was what they were called to do.

#### Sub-theme 9.1: Calling

Even though the pandemic brought about many challenges for the nurses, they still viewed their jobs as a calling and having a greater meaning and purpose. Some of the nurses in this study stated this explicitly:

‘This is definite calling for me, everything I do, I do it with no expectation of getting anything out of it, whether I am seen or not.’ (Participant 11, 64 years old, professional nurse)

#### Sub-theme 9.2: Meaningful work

The participants consistently mentioned having passion and a love for the job. Some asserted that this passion kept them going through the pandemic:

‘Nursing for me is not just a job. I love it and am very passionate about it.’ (Participant 3, 53 years old, professional nurse)

## Discussion

This article focuses on how the COVID-19 pandemic, as a specific event, led to transitions and strain, creating identity demands that led to identity tensions and identity work in nurses during the pandemic. The study found that nurses had to deal with equipment and staff shortages, the lack of social distancing and a lack of recognition. The challenges experienced as a result of the pandemic were felt all over the world; the lack of readiness was cited as a major factor that caused hospitals to have to deal with shortages of personal protective equipment, gloves, masks and insufficient intensive care unit (ICU) beds and ventilators (Kaye et al. 2021:295).

These challenges placed identity demands on nurses during the pandemic. The nurses experienced long working hours, burnout, increased workload and high stress levels, emotional and mental strain and unsafe working conditions. The findings of this study corroborate those of Arasli et al. (2020:2), who found that the COVID-19 pandemic significantly affected nurses’ psychological state, and those of other studies by Yousaf, Nassani and Haffar (2021:955) and Jansoon and Rello (2020:1), which identified the negative effects of the pandemic as mostly because of increased workload and exhaustion. Some nurses were more emotional in certain situations, while others struggled more mentally. In the study by Yang et al. (2022:2861) of nurses’ experiences during the pandemic, they found that nurses had to cope with high stress levels, burnout and symptoms of post-traumatic stress disorder (PTSD) because of not seeing their loved ones for a long time. From this study, many nurses spoke of the need for recognition, acknowledgement and if they had had greater support from management in some way, a bigger difference would have been made.

The identity demands experienced led to identity tensions for the nurses. One such tension was the importance of balancing work demands (workload, long working hours) and personal life. In separating the role of being a nurse (duties and functions) and their personal identity as individuals, the nurses in this study stated that they were human first before being nurses. The pandemic brought different demand-related stresses and work tensions, which changed things for them. Ashforth (2001:32) termed this *situational relevance* and defined it as ‘the degree to which a given identity is socially appropriate to a given situation’; Kreiner et al. (2006:1043) noted this as *setting limits*. Both concepts – situational relevance and setting limits – fit within the framework of this study. In separating their two distinct identities, most of the nurses were able to separate being a nurse from other roles they occupy. Most of the nurses could differentiate between being at work with the requirements needed at the time (showing empathy and treating patients) and leaving everything behind when it was home time, which helped them deal with the demands at hand during the pandemic.

In the study, the aim was also to establish what identity-work strategies these nurses engaged in to deal with their identity demands and tensions. Meaningful work and calling were the driving motives in the nurses’ construction of their identity and purpose, and they used different identity-work modes to engage their identity work. The first mode, namely, the *cognitive mode*, entailed the nurses’ making sense of the new situations and changes brought on by the pandemic; this was linked to the identity-work tactic of recognition (dawning). The cognitive identity-work mode encompasses an individual’s interpreting and making sense of their identity amid varied challenges and tensions (Caza et al. 2018:891). The second mode, that is, *behavioural mode* was also used by the nurses; this encompasses the actions by which people revise and maintain their identities (Caza et al. 2018:893). The nurses invested in family time and outside activities that did not involve nursing. In addition, some nurses turned to prayer, spiritual upliftment and coming together to cope.

Other authors have referred to this identity strategy of seeking religious and spiritual upliftment (Adams & Crafford 2012:6) and tapping spiritual resources (Kreiner et al. 2006:1045) – a form of dealing with difficult situations through living by one’s personal ethical values and beliefs.

This is linked to the identity-work tactics of recognition (dawning) and adapting, both used by some nurses. Upon reaching recognition (dawning), the nurses realised that the situation had changed and that things would be different, and they, therefore, needed to change how they reacted to and handled matters. Another identity-work strategy was upskilling themselves, a behavioural mode of identity work. The nurses were required to upskill themselves in a short space of time to handle new demands created by the pandemic. Kirpal (2004:283) stated that flexibility and mobility enable nurses to adapt to change in their wards and tasks. Some nurses transitioned between roles, for instance, from the maternity ward to the ICU ward and working with new people and teams.

The study also found that nurses experienced episodic identity work triggered by uncertainty and anxiety through the specific events or experiences of the COVID-19 pandemic. Triggers are those events or situations that frustrate or leave more than one identity mode dissatisfied (Lepisto, Crosina & Pratt 2015:18). In a study by Thomas and Linstead (2002:15), episodic identity construction was triggered by an organisational restructure. In this study, the trigger was the changes to how the nurse participants fulfilled their work or roles. Veldhuizen et al. (2021:6) found that owing to the pandemic, the nurses in their study had to change their daily work routines, learn to work with PPE and deal with increased work pressure.

Another main finding of this study revealed that nursing was more than just a job but also a calling, giving the nurses in this study a sense of purpose. Steger et al. (2010:84) described a calling as the belief that God has called one to do a certain job and that it is closely related to meaningful work. Many felt that if they did not experience their work as meaningful, they would have quit their jobs long ago, as that was what kept them going. Meaningful work was defined by Van Wingerden and Van der Stoep (2018:1) as ‘work which is particularly important and holding positive meaning for individuals’. Patience et al. (2020:408) stated that meaningful work is significant for nurses to stay engaged.

### Limitations

The first limitation of this study was that it was conducted during a pandemic, which meant that accessing participants was not easy, as some could not participate in face-to-face interviews because of the risk of being exposed to the virus. The study included participants with different work experiences in various wards and backgrounds, giving different views. However, because of the non-availability of male participants, the sample was limited to only females. In addition, the average age of the participants is mid-30s to early-60s, with people with a strong tenure in their fields, whereas perhaps a younger demographic would have provided a different view and impacted on the findings, as they would still have been very new in their careers.

### Implications for hospitals and nurses

Nurses’ commitment to their jobs, sense of purpose and meaningful work increased during the pandemic. Although challenges were experienced, a constant love for their job and their patients was evident across the entire study. The experiences outlined by the nurses brought on by the pandemic (lack of recognition, having to upskill, working long hours, high stress levels, emotional and mental demands) highlighted the need for strategies to be implemented around increased recognition of and support for mental and emotional challenges to ensure the overall health of nurses. Hospital management should include various interventions and strategies, such as counselling programmes, to assist nurses in dealing with the physical and psychological effects associated with pandemics and epidemics and overall dealing with challenges within hospitals.

## Conclusion

The COVID-19 pandemic brought to light the broken state of our healthcare facilities and how changes need to be implemented. The main aim of this study was to establish how the challenges experienced during the COVID-19 pandemic may have negatively influenced the way nurses identified with their tasks and roles. The main finding was that the combination of meaningful work and having a calling were fundamental for nurses during the pandemic, and that identity-work tactics and strategies helped deal with all the new work demands and tensions that came about because of the pandemic. It also identified that meaningful work and a calling are a priority for nurses. Nurses have long been serving communities and, in moving forward, the government should focus more on their wellbeing, showing them support and recognising their efforts.

## Appendix 1: Semi-structured Interview Guide

### Interview questions

#### Explanation of the study

The study is about identity work of nurses in public hospitals. (How they experience their roles, jobs and how they have managed to maintain their identities during the coronavirus crisis.) There are no right or wrong answers and you can decide to withdraw from the study at any time. You will remain anonymous and the recordings and transcriptions will be kept safe and will not be shared with anyone. Interviews will be conducted at a time, which is convenient for the nurses to avoid disruptions to their normal working hours, and the duration of the interviews will approximately last for 45 min.

#### Semi-structured interview questions:

This interview is being conducted to find out about your background and career and to determine how the coronavirus crisis has influenced your work and working environment. Open-ended questions will be asked to gain an understanding of the views of the participants during this crisis. Informed consent forms will be given to each participant explaining the full process and purpose of the study and that participation is completely voluntary, and if at any point, they want to withdraw from the study they may.

All recordings will be kept safe, and only the researchers involved with the study will have access to the data; all information shared via email will also be password-protected, and all written data will be stored for 5 years at the researcher’s house and then disposed of, and electronic data will be stored for an indefinite period, but interview recordings will be disposed of once no longer required.

1. Tell me a bit about yourself? Your background and why you chose this career path? What led you to become a nurse? What do you like the most and least about your job and how would you describe the role you play in this hospital?
2. Why did you choose to become a nurse? What are your primary responsibilities as a nurse? Briefly describe your role and daily tasks and what your typical day as a nurse looks like? What are some of the challenges you experience when it comes to doing your job and what measures have you implemented to deal with them?
3. What is the impact of the coronavirus pandemic on your job (as a nurse) and how has the current pandemic impacted the way in which you do things? What are some of the challenges that you have faced thus far during this time, and how have you coped with them?
4. How are you currently experiencing your work environment in the midst of the coronavirus crisis? If you had to implement certain strategies to better cope with the current situation what would it be? What do you think can be done to better improve nursing care and service delivery during the current pandemic?
5. How are you dealing with the current demands, stress and work tensions associated with the job during this pandemic?
6. What tensions and demands (identity threats) are you experiencing in your current work environment and what strategies have you deployed to better cope?
7. Would you say being a nurse is much more than just fulfilling a role but can also be viewed as a calling and a part of who you are as a person? And at which point did you come to realise this?
8. Are there times when you have felt that the job is demanding too much from you or you have had to change certain aspects of yourself in order to better deal with the emotional and mental demands of the job especially during the current pandemic? How have you managed to overcome this?
9. Was there any other job or career path you wanted to pursue outside of nursing and what was it?

## References

[CIT0001] Adams, B.G. & Crafford, A., 2012, ‘Identity at work: Exploring strategies for identity work’, *SA Journal of Industrial Psychology* 38(1), 1–11. 10.4102/sajip.v38i1.904

[CIT0002] Al-Ababneh, M.M., 2020, ‘Linking ontology, epistemology and research methodology’, *Science & Philosophy* 8(1), 75–91. 10.23756/sp.v8i1.50

[CIT0003] Almomani, M., Khater, W.A., Akhu-Zaheya, L.M., Alloubani, A., AlAshram, S.A., Azab, M. et al., 2022, ‘Nurses’ experiences of caring for patients with COVID-19: A qualitative study’, *SAGE Open* 12(4), 1–15. 10.1177/21582440221144982 PMC9791001 36588664

[CIT0004] Arasli, H., Furunes, T., Jafari, K., Saydam, M.B. & Degirmencioglu, Z., 2020, ‘Hearing the voices of wingless angels: A critical content analysis of nurses’ covid-19 experiences’, *International Journal of Environmental Research and Public Health* 17(22), 1–16. 10.3390/ijerph17228484 PMC7696738 33207740

[CIT0005] Ashforth, B.E., 2001, *Role transitions in organizational life: An identity-based perspective*, Erlbaum, Mahwah, NJ.

[CIT0006] Braun, V. & Clarke, V., 2006, ‘Using thematic analysis in psychology’, *Qualitative Research in Psychology* 3(2), 77–101. 10.1191/1478088706qp063oa

[CIT0007] Caza, B.B., Vough, H. & Puranik, H., 2018, ‘Identity work in organizations and occupations: Definitions, theories, and pathways forward’, *Journal of Organizational Behavior* 39(7), 889–910. 10.1002/job.2318

[CIT0008] Jansoon, M. & Rello, J., 2020, ‘iMedPub journals mental health in healthcare workers and the covid-19 pandemic era : Novel challenge for critical care abstract’, *Journal of Intensive and Critical Care* 6, 1–3. 10.36648/2471-8505.6.2.6

[CIT0009] Kaye, A.D., Okeagu, C.N., Pham, A.D., Silva, R.A., Hurley, J.J., Arron, B.L. et al., 2021, ‘Economic impact of COVID-19 pandemic on healthcare facilities and systems: International perspectives’, *Best Practice and Research: Clinical Anaesthesiology* 35(3), 293–306. 10.1016/j.bpa.2020.11.009 34511220 PMC7670225

[CIT0010] Kirpal, S., 2004, ‘Work identities of nurses between caring and efficiency demands’, *Career Development International* 9(3), 274–304. 10.1108/13620430410535850

[CIT0011] Kreiner, G.E., Hollensbe, E.C. & Sheep, M.L., 2006, ‘Where is the “me” among the “we”? Identity work and the search for optimal balance’, *Academy of Management Journal* 49(5), 1031–1057. 10.5465/AMJ.2006.22798186

[CIT0012] Lepisto, D.A., Crosina, E. & Pratt, M.G., 2015, ‘Identity work within and beyond the professions: Toward a theoretical integration and extension’, in A. Desilva & M. Aparicio (eds.), *International Handbook of Professional Identities*, pp. 11–37, Scientific & Academic Publishing, Rosemead, CA.

[CIT0013] Manyisa, Z.M. & Van Aswegen, E.J., 2017, ‘Factors affecting working conditions in public hospitals: A literature review’, *International Journal of Africa Nursing Sciences* 6(1), 28–38. 10.1016/j.ijans.2017.02.002

[CIT0014] Muller, A.E., Hafstad, E.V., Himmels, J.P.W., Smedslund, G., Flottorp, S., Stensland, S.Ø. et al., 2020, ‘The mental health impact of the covid-19 pandemic on healthcare workers, and interventions to help them: A rapid systematic review’, *Psychiatry Research* 293, 1–11. 10.1016/j.psychres.2020.113441 PMC7462563 32898840

[CIT0015] Patience, M.G., De Braine, R. & Dhanpat, N., 2020, ‘Job demands, job resources, and work engagement among South African nurses’, *Journal of Psychology in Africa* 30(5), 408–416. 10.1080/14330237.2020.1821315

[CIT0016] Steger, M.F., Pickering, N.K., Shin, J.Y. & Dik, B.J., 2010, ‘Calling in work: Secular or sacred?’, *Journal of Career Assessment* 18(1), 82–96. 10.1177/1069072709350905

[CIT0017] Thomas, R. & Linstead, A., 2002, ‘Losing the plot? Middle managers and identity’, *Organization* 9(1), 71–93. 10.1177/135050840291004

[CIT0018] Van Manen, M., 2017, ‘Phenomenology in its original sense’, *Qualitative Health Research* 27(6), 810–825. 10.1177/1049732317699381 28682720

[CIT0019] Van Wingerden, J. & Van der Stoep, J., 2018, ‘The motivational potential of meaningful work: Relationships with strengths use, work engagement, and performance’, *PLoS One* 13(6), 1–11. 10.1371/journal.pone.0197599 PMC5999113 29897935

[CIT0020] Veldhuizen, J.D., Zwakhalen, S., Buurman, B.M. & Bleijenberg, N., 2021, ‘The impact of COVID-19 from the perspectives of Dutch district nurses: A mixed-methods study’, *International Journal of Environmental Research and Public Health* 18(24), 13266. 10.3390/ijerph182413266 34948875 PMC8703809

[CIT0021] Yang, B.J., Yen, C.W., Lin, S.J., Huang, C.H., Wu, J.L., Cheng, Y.R. et al., 2022, ‘Emergency nurses’ burnout levels as the mediator of the relationship between stress and posttraumatic stress disorder symptoms during COVID-19 pandemic’, *Journal of Advanced Nursing* 78(9), 2861–2871. 10.1111/jan.15214 35307874 PMC9111628

[CIT0022] Yousaf, Z., Nassani, A.A. & Haffar, M., 2021, ‘Destructive role of COVID-19 fear on nurses performance: Mediating role of stress’, *Nursing Reports* 11(4), 955–964. 10.3390/nursrep11040087 34968281 PMC8715474

